# An early predictive model of frailty for older inpatients according to nutritional risk: protocol for a cohort study in China

**DOI:** 10.1186/s12877-021-02396-3

**Published:** 2021-08-18

**Authors:** Hongpeng Liu, Jing Jiao, Minglei Zhu, Xianxiu Wen, Jingfen Jin, Hui Wang, Dongmei Lv, Shengxiu Zhao, Wei Chen, Xinjuan Wu, Tao Xu

**Affiliations:** 1 Department of Nursing, Chinese Academy of Medical Sciences - Peking Union Medical College, Peking Union Medical College Hospital (Dongdan campus), No.1 Shuaifuyuan Wangfujing Dongcheng District, 100730 Beijing, China; 2 Department of Geriatrics, Chinese Academy of Medical Sciences - Peking Union Medical College, Peking Union Medical College Hospital (Dongdan campus), No.1 Shuaifuyuan Wangfujing Dongcheng District, 100730 Beijing, China; 3 Department of Nursing, Sichuan Provincial People’s Hospital, No.32 West Second Section First Ring Road, 610072 Chengdu, China; 4 Department of Nursing, The Second Affiliated Hospital, Zhejiang University School of Medicine, 88 Jiefang Road, 310009 Hangzhou, China; 5 Department of Nursing, Tongji Hospital, Tongji Medical College, Huazhong University of Science and Technology, 1037 Luoyu Road, Hongshan District, 430074 Wuhan, China; 6 Department of Nursing, The Second Affiliated Hospital of Haerbin Medical University, 246 Xuefu Road, 150081 Haerbin, China; 7 Department of Nursing, Qinghai Provincial People’s Hospital, 2 Gonghe Road, Chengdong District, 810007 Xining, China; 8 Department of Clinical Nutrition, Department of Health Medicine, Chinese Academy of Medical Sciences - Peking Union Medical College, Peking Union Medical College Hospital, (Dongdan campus), No.1 Shuaifuyuan Wangfujing Dongcheng District, 100730 Beijing, China; 9 Beijing Key Laboratory of the Innovative Development of Functional Staple and the Nutritional Intervention for Chronic Disease, Building 6, No. 24 Courtyard, Jiuxianqiao Middle Road, Chaoyang District, 100015 Beijing, China; 10 Department of Epidemiology and Statistics, Institute of Basic Medical Sciences, School of Basic Medicine, Chinese Academy of Medical Sciences, Peking Union Medical College, 5 Dongdan Santiao, Dongcheng District, 100005 Beijing, China

**Keywords:** Nutritional status screening, Malnutrition parameters, Mortality, Length of stay, Readmission, Older inpatients

## Abstract

**Background:**

Previous reports suggest that the attributes of frailty are multidimensional and include nutrition, cognition, mentality, and other aspects. We aim to develop an early warning model of frailty based on nutritional risk screening and apply the frailty early warning model in the clinic to screen high-risk patients and provide corresponding intervention target information.

**Methods:**

The proposed study includes two stages. In the first stage, we aim to develop a prediction model of frailty among older inpatients with nutritional risk. Study data were collected from a population-based aging cohort study in China. A prospective cohort study design will be used in the second stage of the study. We will recruit 266 older inpatients (age 65 years or older) with nutritional risk, and we will apply the frailty model in the clinic to explore the predictive ability of the model in participants, assess patients’ health outcomes with implementation of the frailty model, and compare the model with existing frailty assessment tools. Patients’ health outcomes will be measured at admission and at 30-day follow-up.

**Discussion:**

This project is the first to develop an early prediction model of frailty for older inpatients according to nutritional risk in a nationally representative sample of Chinese older inpatients of tertiary hospitals. The results will hopefully help to promote the development of more detailed frailty assessment tools according to nutritional risk, which may ultimately lead to reduced health care costs and improvement in independence and quality of life among geriatric patients.

**Trial registration:**

Chinese Clinical Trial Registry, ChiCTR1800017682, registered August 9, 2018; and ChiCTR2100044148, registered March 11, 2021.

**Supplementary Information:**

The online version contains supplementary material available at 10.1186/s12877-021-02396-3.

## Background

With the rapid rate of change in health services and the global economy, the world’s population is experiencing increased longevity, with increases in the segment of the global population comprising older adults [1–3]. Epidemiologic studies show that the proportion of adults aged 65 years and older is expected to exceed 16 % of the world’s population by 2050 [4], with 80 % living in low- and middle-income countries [5]. As the second largest economy worldwide, China currently has the world’s largest population with 1.44 billion people, which accounts for 19 % of the global population, and China is swiftly changing into an aging country [3, 6–8].

Undernutrition is a frequent and serious condition within the geriatric population admitted to the hospital, and nutritional status often deteriorates further during hospitalization [8–11]. Therefore, during hospital admission or at discharge, a large number of older patients will still be malnourished or at risk of malnutrition [12, 13]. As per the American Society for Parenteral and Enteral Nutrition recommendation, malnutrition refers to all deviations from adequate and optimal nutritional status, including energy undernutrition and overnutrition [14]. The term undernutrition is used to refer to generally poor nutritional status; however, because malnutrition often refers to undernutrition, both terms are widely used in a similar sense [15, 16]. Previous studies have reported that the prevalence of hospital malnutrition or malnutrition risk in older inpatients is high (30–50 %) [10, 17, 18]. Its negative impact on health substantially affects quality of life by increasing the risk of frailty, disability, and mortality [19, 20].

Poor nutritional status can increase the age-associated loss of muscle mass and strength and is therefore seen as having an important role in the development of sarcopenia and subsequent physical impairment [21], which both represent substantial elements of the frailty syndrome [16, 22, 23]. Boulos et al. conducted a cross-sectional study among 1200 community–dwelling older adults living in a rural setting of Lebanon to examine the association between malnutrition and frailty. Those authors reported that both malnutrition and malnutrition risk were related to a significantly increased risk of frailty (odds ratio [OR] 3.72, 95 % confidence interval [CI] 1.40 to 9.94 and OR 3.66, 95 % CI 2.32 to 5.76, respectively) [9].

Frailty is a complex, age-related clinical condition characterized by a decline in physiological capacity across several organ systems, and it is a state of increased vulnerability to stress [9, 22, 24]. However, older adults with frailty have an increased likelihood of unmet care needs, falls and fractures, hospital readmissions, increased length and cost of hospital stay, lower quality of life, iatrogenic complications, and early mortality [10, 24]. This increased risk of adverse clinical outcomes can occur even without the presence of comorbidities [24, 25].

The investigation of frailty has attracted enormous scientific interest in the past few years as it affects multiple domains of human functioning, including nutritional status, cognitive function, gait, mobility, balance, muscle strength, endurance, and activities of daily living (ADL) [26]. Additionally, previous reports have suggested that the attributes of frailty are multidimensional and that its definition should comprise nutrition, cognition, mentality, and other aspects [27–30]. Identification is a critical step in the intervention or management of frailty [24, 29, 30]. Both the cycle of frailty model [22] and the integral conceptual frailty model [31] include nutrition as a factor to explain frailty. However, undernutrition is considered a sub-factor in physical frailty in the integral conceptual frailty model and a predisposing factor for frailty in the cycle of frailty model [22]. Previous studies have mainly investigated the relationship between undernutrition and frailty [9, 21, 32, 33], but none have reported the degree of influence on frailty according to nutritional risk, despite nutritional risk being a modifiable variable.

Using the current study protocol, in the first stage of this study, we used data from a cohort study to develop an early warning model of frailty based on nutritional risk screening. In the second stage of the study, we report the prevalence of frailty among older inpatients with nutritional risk and apply the frailty early warning model in the clinic to screen high-risk patients and provide corresponding intervention target information.

## Methods

### Study design

The study includes two stages. In the first stage, we aim to develop a prediction model of frailty among older inpatients with nutritional risk. Study data were collected from a population-based aging cohort study in China. A prospective cohort study design will be used in the second stage of the study. We will apply the frailty model in the clinic to explore the predictive ability of the model in participants, assess patients’ health outcomes with implementation of the frailty model, and compare the model with existing frailty assessment tools.

### Developing the frailty model according to nutritional risk

#### Data used for modeling

The data used for development of the prediction model were derived from an ongoing, prospective large-scale cohort study among older Chinese inpatients at tertiary hospitals (Chinese Clinical Trial Registry Number: ChiCTR1800017682). In the present study, we used baseline survey data collected from October 2018 to February 2019; details can be found elsewhere [3, 10]. Briefly, this study is a nationwide survey that provides representative data for the investigation of geriatric factors, such as nutritional status, cognition, or physical activity, in hospitalized older individuals aged ≥ 65 years nationwide. Eligible participants are recruited from five provinces and one municipality in China (southwest: Sichuan Province; northeast: Heilongjiang Province; south–central: Hubei Province; northern: Beijing municipality/city; northwest: Qinghai Province; eastern: Zhejiang Province). All eligible older individuals are continuously enrolled. Surveys are administered by trained nurse interviewers using a structured questionnaire. The interview language used is standard Mandarin/Putonghua. Proxy respondents, usually a spouse or other legal guardian, are interviewed if the patient is unable to answer the questions themselves.

#### Feasibility of recruitment and sample size

In the first stage, for the sample size required for modeling, we referred to the method of estimating sample size for multivariate logistic regression. For the categories with a smaller proportion of the outcome variables, the sample size should be at least 10 times the number of independent variables. There are two categories of dependent variables in this study (no frailty and frailty), and 20 important independent variables were initially estimated. Therefore, the sample size of the intervention group in this study is at least 200 patients. According to the literature data, the incidence of frailty in the older population with nutritional risk is approximately 26.5–54 % [18, 32, 34, 35]. Taking into account an expected incidence of 40 % and loss to follow-up of 10–20 %, we aim to include 600 patients. As for the total sample size, with reference to logistic regression modeling requirements, the modeling sample size is approximately two-thirds of the total sample size and the model verification sample size is approximately one-third of the total sample size. The total sample size is calculated to be at least 900 patients, of which at least 300 patients will be used for model verification.

#### Population and inclusion and exclusion criteria

In the first stage of modeling, participants must meet the following criteria: having no frailty according to the FRAIL (Fatigue, Resistance, Ambulation, Illnesses, & Loss of Weight) scale (scores from 0 to 2), malnourished or at risk of malnutrition according to the Mini-Nutritional Assessment-Short Form (MNA-SF; scores range from 0 to 11), and written informed consent provided by patients enrolled in this study.

Exclusion criterion were as follows: patients with frailty at the time of enrollment; patients who are persistently unconsciousness or unable to provide informed consent for participation, or their caregivers were unable to provide effective information; patients who were initially admitted to the intensive care unit (ICU) [13]; patients with anorexia nervosa, acute pancreatitis, acute liver failure, cystic fibrosis, stem cell transplantation, severe chronic gastrointestinal diseases, acute infectious diseases or chronic wasting diseases at enrollment; and patients who were lost to follow-up or had died at the 30-day of follow-up. A total of 3027 patients were enrolled in the current study.

#### Definition of covariates

Potentially associated factors of frailty in the models included [16, 36–43] age, sex, ethnicity, education level, marital status, body mass index (BMI), living alone, living conditions, smoking, alcohol consumption, falling accidents in the past 12 months, immobilization for more than 4 weeks, polypharmacy, fatigue, resistance (ability to climb stairs), ambulation (ability to walk 100 m), illnesses (> 5), loss of more than 5 % body weight, nutritional risk, ADL, instrumental ADL, depression, cognitive function, handgrip strength, vision, hearing, sleeping, urinary function, and defecation function.

Age was grouped as 65–74 years old, 75–85 years old, and 85 years old and above. Ethnicity was categorized as Han and ethnic minorities. Education level was categorized as illiterate, primary school, junior high school, and high school and above. Marital status was categorized as married and divorced or widowed. Living alone was categorized as living alone or not living alone [40]. Living conditions was classified as living in a building with an elevator, a building without an elevator, or a bungalow. Polypharmacy was categorized as 0, 1–2, 3–4, 5–6, and more than 7 [16].

BMI was calculated as body weight divided by height (in meters) squared (kg/m^2^) [18] and was used to classify patients into groups of < 19 kg/m^2^, 19 to < 21 kg/m^2^, 21 to < 23 kg/m^2^, and ≥ 23 kg/m^2^. Participants’ weight in kg was measured to the nearest 0.1 kg using a digital electronic chair scale, and height (in cm) was measured to the nearest 1 mm using a stadiometer. Study participants were weighed while wearing light clothing and no shoes.

We referred to the FRAIL scale [38, 44], such as the items of fatigue, resistance (ability to climb stairs), ambulation (ability to walk 100 m), illnesses (> 5), and loss of more than 5 % body weight, which has been validated in Chinese older adults [45].

Nutritional risk was measured using the MNA-SF, a six-item scale with scores ranging from 0 to 14 points [19]. Patients were categorized into patients at risk of malnutrition (8–11 points) or malnourished (0–7 points) [39]. The MNA-SF has been validated in the Chinese population and has excellent test characteristics [10, 46].

ADL were measured using the Barthel Index, which is a 10-item instrument measuring disability in terms of a person’s level of functional independence in personal ADL [47–49]. A higher score means better capacity to perform daily living activities [48, 49]. Patients were categorized into those with a score < 75 and ≥ 75.

Instrumental ADL were measured using the Instrumental Activities of Daily Living Scale [50], which includes a range of higher-level activities that are considered to address the capacity of older adults to interact with their community [51]. The scores on this scale range from 0 to 8, with 0 being the least independent and 8 being the most independent [50, 52, 53]. Patients were categorized into groups with scores of < 6 and ≥ 6 on the eight-item scale.

The depression assessment scale was developed on the basis of the Geriatric Depression Scale 15 (GDS15) [54], with a higher score denoting more severe depression. Patients were categorized into groups with scores of 0–5 and 6–15.

Assessment of cognitive function was on the basis of the Mini-Cog [55, 56] and was dichotomized as normal cognitive function (scores of 3–5) and cognitive dysfunction (scores 0–2).

Handgrip strength was categorized as normal (greater than 28 kg in men and greater than 18 kg in women) and abnormal, according to the Asian Working Group for Sarcopenia in 2019 [57].

#### Outcomes

The dependent (outcome) variable is frailty, defined as “multi-systemic functional decline below a certain level, leading to increased vulnerability to a minor stressor with poor outcomes of disability and/or mortality” [16, 58]. The outcome will be expressed using three categories: non-frail, pre-frail, and frail when meeting 0, 1 or 2, and ≥ 3 criteria of the FRAIL scale. A larger total score indicates more a severely frail condition.

#### Statistical considerations

Descriptive results are expressed as mean and standard deviation (SD) or as number and percentage. Bivariate analyses will be performed using the χ^2^ test or Fisher’s exact test for qualitative variables and the Student *t-*test, analysis of variance (ANOVA), or Kruskal–Wallis test for quantitative variables. Logistic regression analysis will apply in variable selection and we used an entry criterion of *P* < 0.05. The data set will be divided into a training (70 %) and verification (30 %) set using random sampling. We will establish a frailty prediction model using the modeling data set, and discrimination will be expressed using area under the receiver operating curve and the Hosmer–Lemeshow test to evaluate goodness of fit [59]. As for internal validation, we will use a bootstrap technique with 1000 resamples from the training data set. The verification data set will be used for external verification, and its effectiveness evaluated according to accuracy and area under the receiver operating curve. All statistical analysis will be performed using Stata version 14 for Windows (Stata Corp, College Station, TX, USA). A *P* value of less than 0.05 will be considered statistically significant.

#### Bioethics

The first stage of the study was conducted according to the ethical principles established in the Declaration of Helsinki. The Ethics Committee of Peking Union Medical College Hospital (S-K540) approved the protocol. Written informed consent was provided by all patients enrolled in this study.

### Applying the frailty prediction model in the clinic

#### Population and inclusion and exclusion criteria

All older patients (age 65 years or more, BI scores ≥ 75 points, and estimated survival time > 3 months) who were hospitalized for minimum of 4 days in the wards of Peking Union Medical College Hospital, will be screened by a research assistant using the frailty prediction model. The other inclusion criteria are the same as in the first stage of the study. In the second stage, we will also exclude patients who were included in model development during the first stage.

#### Feasibility of recruitment and sample size

In the second stage, a previous study suggested that the relative risk of readmission is 1.9 among the malnourished older patients compared with the robust [60], and the hospital readmission rate among healthy older people with nutritional risk is 17 %. Taking into account an expected refusal rate of 20 %, we aim to include two groups of 266 patients, to be reached in approximately 6 months.

#### Procedure

After obtaining participants’ informed consent, an inventory will be made of possible confounders. This includes the following: sociodemographic data (age, sex, ethnicity, marital status, education level, type of insurance), hospital admissions, medical diagnosis, living conditions, smoking, and alcohol consumption.

Anthropometric indicators will include admission to the following. (1) Standing height; in the case of bedridden patients, the formula of Chumlea will be used to estimate height [61]. (2) Weight. (3) BMI. (4) Handgrip strength in kg, measured with a hydraulic hand dynamometer (EH101; Camry, Guangdong Province, China)  [62]. Patients will be seated with forearms resting on the arms of a chair and asked to perform three maximum force trials with their dominant hand, using the second handle position. The maximum grip score among the three values will be used [63]. Nutritional risk will be measured using MNA-SF score (0–11 points ranging from “malnourished” to “at risk of malnutrition”) [10]. For frailty assessment, the Fried frailty phenotypes (FPs) [22] will be adopted as the “standard” for comparisons with the frailty model developed in the first stage of the current study. The five phenotypes of frailty that will be used include poor appetite, exhaustion, low physical activity, poor walking ability, and poor twisting ability of fingers. Participants are to be classified as non-frail, pre-frail, and frail when meeting 0, 1 or 2, and ≥ 3 criteria [43].

To determine the degree of dependency, the BI will be used to perform physical function assessment (basic ADL) among patients. Data collection will be carried out by direct observation or by asking the patient, if possible. Scores range from 0 to 100, with a higher score indicating greater independence.

Biochemical markers include serum albumin and prealbumin (colorimetry), hemoglobin and hematocrit (fluorescence and optical methods), cholesterol (enzymatic techniques), and myokines (enzyme-linked immunosorbent assay (ELISA); Shanghai Enzyme-Linked Biotechnology Co., Ltd.).

#### Outcome parameters

Health outcomes will be measured at admission and at 30-day follow-up. The composite primary endpoint includes adverse clinical outcomes within 30 days: non-elective hospital readmission after discharge (second and subsequent hospitalizations during the period analyzed), frailty, all-cause mortality (all-cause mortality recorded at 30 days, including in-hospital deaths), ICU admission, a decline in functional status of 10 % or more from admission to day 30 as measured with the BI, and major complications as a new occurrence including adjudicated diagnosis of nosocomial infection, respiratory failure, cardiovascular event (i.e., stroke, intracranial bleeding, cardiac arrest, myocardial infarction, pulmonary embolism), acute renal failure, and gastrointestinal failure (i.e., hemorrhage, intestinal perforation, acute pancreatitis) [13].

The main secondary endpoints include length of hospital stay (LoS; duration of hospitalization, number of hospitalization days), health-related quality of life as measured using the three-level EuroQol five-dimensions (EQ-5D-3 L) questionnaire; index values range from 0 to 1, with higher scores indicating better quality of life, including the self-assessment visual analogue scale (EQ-5D VAS; scores range from 0 to 100, with higher scores indicating better health status).

#### Statistical analysis

Continuous variables are described using mean and SD or median with interquartile range in the case of a skewed distribution. Categorical variables are described as number and percentage. ANOVA will be used to examine the statistical differences in variables among different groups. We will perform bivariate analyses using the χ^2^ test or Fisher’s exact test for qualitative variables and Kruskal–Wallis test for quantitative variables. Cox proportional hazards models will be constructed to determine the association of frailty score at baseline with mortality. Multiple linear regression models will be applied to evaluate the relationship between baseline frailty score and health clinical outcomes. We will use the Kappa statistic to evaluate the consistency of the new frailty prediction model and FP. The correlation between biochemical markers and frailty will be assessed using Pearson’s correlation coefficient. All statistical analysis will be performed using Stata version 14 for Windows (Stata Corp, College Station, TX, USA). A *P* value of less than 0.05 is considered statistically significant.

#### Organization and quality control

The data will be gathered by the primary investigator and research assistants. The primary investigator is responsible for the informed consent procedure, final participant selection, measurements, analysis, and reports. The primary investigator will be assisted by two research assistants. Data flow will be controlled by the primary investigator. Data entry and control will be conducted by the research assistants under supervision of the investigator. The primary investigator is responsible for the data cleaning and analysis.

#### Consent and ethics

This second stage of the study was approved by the Ethics Committee of Peking Union Medical College Hospital (JS-2781). All patients will be asked to provide their written informed consent to participate. All procedures of this study are performed in accordance with the principles laid down in the Declaration of Helsinki.

## Discussion

This project is the first to develop an early prediction model of frailty for older inpatients according to nutritional risk in a nationally representative sample of older Chinese inpatients in tertiary hospital.

In the past two decades, Fried et al. [22], Mitnitski et al. [64], Rockwood et al. [65] and other researchers [36, 66] have made great contributions to the measurement tools available for identifying frailty. These include the physical phenotype model of Fried et al. [22] and the FRAIL scale [36], the deficit accumulation models of Mitnitski [64] and Rockwood [65], which capture multimorbidity; and mixed physical and psychosocial models, such as the Tilburg Frailty Indicator [66] and Edmonton Frailty Scale [67]. However, the theoretical basis, evaluation items, evaluation methods, and applicable objects differ among these assessment tools. Additionally, none of these tools have been developed on the basis of the Asia-Pacific region, which has the largest population of older adults worldwide combined with large heterogeneity regarding population socioeconomics, provision of health care services, and ethnic diversity [58], especially among the aging Chinese population [6–8].

Many researchers have indicated that frailty is multidimensional and that its definition should comprise nutrition, cognition, mentality, ADL, and other aspects [26–30, 58]. Therefore, using this protocol, we aim to adopt findings and assessment tools from previous studies, and to develop a frailty prediction model according to nutritional risk. Early assessment to identify frailty or pre-frailty is critical in older inpatients and may help in targeting interventions to address and reduce adverse clinical outcomes and improve patient quality of life.

The limitations of this study include the older inpatients enrolled in the first stage of our study were selected from tertiary hospitals and from only one hospital in each province or municipality/city, which may limit the generalizability of our results to different settings. Additionally, the estimated sample size is not sufficiently large in the second stage of the study (*n* = 266).

It is important to develop an early prediction model of frailty for older inpatients according to nutritional risk for the Chinese population, which will provide early warning information for older patients admitted to the hospital. Identifying pre-frailty as early as possible will help to avoid physical dysfunction, hospital readmission, and mortality. The results of this research will help to promote the development of more detailed frailty assessment tools focused on nutritional risk, cognition, depression, and other important factors, which may ultimately lead to reduced health care costs and improvement in mobility, independence, and quality of life among geriatric patients with nutritional risk.

## Supplementary Information

**Additional file 1.**

## Data Availability

Not applicable.

## Abbreviations

MNA-SF: Mini Nutritional Assessment Short-Form
BMI: Body mass index
SD: Standard deviation
CI: Confidence interval
ICU: Intensive care unit
FP: Fried Phenotype
LoS: Length of Hospital Stay
EQ-5D-3L: three-level EuroQol five-dimensional questionnaire
ADL: Activities of Daily Living Scale

## References

[1] The Lancet. Global elderly care in crisis. Lancet 2014;383:927. 10.1016/S0140-6736(14)60463-3 24629279

[2] Bloom DE, Luca DL: Chap. 1 - The Global Demography of Aging: Facts, Explanations, Future. In Handbook of the Economics of Population Aging. Edited by Piggott J, Woodland A: North-Holland; 2016:1;3–56.

[3] Liu H, Jiao J, Zhu C, Zhu M, Wen X, Jin J, Wang H, Lv D, Zhao S, Wu X, Xu T (2020). Potential associated factors of functional disability in Chinese older inpatients: a multicenter cross-sectional study. BMC Geriatr.

[4] Søreide K, Wijnhoven BP (2016). Surgery for an ageing population. Br J Surg.

[5] The L. How to cope with an ageing population. Lancet 2013;382:1225. 10.1016/S0140-6736(13)62080-2 24120186

[6] Lv X, Li W, Ma Y, Chen H, Zeng Y, Yu X, Hofman A, Wang H (2019). Cognitive decline and mortality among community-dwelling Chinese older people. BMC Med.

[7] Fang EF, Scheibye-Knudsen M, Jahn HJ, Li J, Ling L, Guo H, Zhu X, Preedy V, Lu H, Bohr VA (2015). A research agenda for aging in China in the 21st century. Ageing Res Rev.

[8] The L. Ageing and health—an agenda half completed. Lancet 2015;386:1509. 10.1016/S0140-6736(15)00521-8 26530601

[9] Boulos C, Salameh P, Barberger-Gateau P (2016). Malnutrition and frailty in community dwelling older adults living in a rural setting. Clin Nutr.

[10] Liu H, Jiao J, Zhu C, Zhu M, Wen X, Jin J, Wang H, Lv D, Zhao S, Wu X, Xu T. Associations Between Nutritional Status, Sociodemographic Characteristics, and Health-Related Variables and Health-Related Quality of Life Among Chinese Elderly Patients: A Multicenter Prospective Study. Front Nutri. 2020;7:583161. 10.3389/fnut.2020.583161 PMC7596354 33178722

[11] McWhirter JP, Pennington CR (1994). Incidence and recognition of malnutrition in hospital. BMJ.

[12] Jie B, Jiang ZM, Nolan MT, Efron DT, Zhu SN, Yu K, Kondrup J (2010). Impact of nutritional support on clinical outcome in patients at nutritional risk: a multicenter, prospective cohort study in Baltimore and Beijing teaching hospitals. Nutrition.

[13] Schuetz P, Fehr R, Baechli V, Geiser M, Deiss M, Gomes F, Kutz A, Tribolet P, Bregenzer T, Braun N (2019). Individualised nutritional support in medical inpatients at nutritional risk: a randomised clinical trial. Lancet.

[14] Soeters PB, Schols AM (2009). Advances in understanding and assessing malnutrition. Curr Opin Clin Nutr Metab Care.

[15] White JV, Guenter P, Jensen G, Malone A, Schofield M (2012). Consensus statement: Academy of Nutrition and Dietetics and American Society for Parenteral and Enteral Nutrition: characteristics recommended for the identification and documentation of adult malnutrition (undernutrition). JPEN J Parenter Enteral Nutr.

[16] Kim E, Sok SR, Won CW (2021). Factors affecting frailty among community-dwelling older adults: A multi-group path analysis according to nutritional status. Int J Nurs Stud.

[17] Vanderwee K, Clays E, Bocquaert I, Gobert M, Folens B, Defloor T (2010). Malnutrition and associated factors in elderly hospital patients: a Belgian cross-sectional, multi-centre study. Clin Nutr.

[18] Martínez-Reig M, Gómez-Arnedo L, Alfonso-Silguero SA, Juncos-Martínez G, Romero L, Abizanda P (2014). Nutritional risk, nutritional status and incident disability in older adults. The FRADEA study. J Nutr Health Aging.

[19] Valmorbida E, Trevisan C, Imoscopi A, Mazzochin M, Manzato E, Sergi G (2020). Malnutrition is associated with increased risk of hospital admission and death in the first 18 months of institutionalization. Clin Nutr.

[20] Khalatbari-Soltani S, Marques-Vidal P (2016). Impact of nutritional risk screening in hospitalized patients on management, outcome and costs: A retrospective study. Clin Nutr.

[21] Bollwein J, Volkert D, Diekmann R, Kaiser MJ, Uter W, Vidal K, Sieber CC, Bauer JM (2013). Nutritional status according to the mini nutritional assessment (MNA®) and frailty in community dwelling older persons: a close relationship. J Nutr Health Aging.

[22] Fried LP, Tangen CM, Walston J, Newman AB, Hirsch C, Gottdiener J, Seeman T, Tracy R, Kop WJ, Burke G, McBurnie MA (2001). Frailty in older adults: evidence for a phenotype. J Gerontol A Biol Sci Med Sci.

[23] Chang SF (2017). Frailty Is a Major Related Factor for at Risk of Malnutrition in Community-Dwelling Older Adults. J Nurs Scholarsh.

[24] Dent E, Martin FC, Bergman H, Woo J, Romero-Ortuno R, Walston JD (2019). Management of frailty: opportunities, challenges, and future directions. Lancet.

[25] Fried LP, Ferrucci L, Darer J, Williamson JD, Anderson G (2004). Untangling the concepts of disability, frailty, and comorbidity: implications for improved targeting and care. J Gerontol A Biol Sci Med Sci.

[26] Fan J, Yu C, Guo Y, Bian Z, Sun Z, Yang L, Chen Y, Du H, Li Z, Lei Y (2020). Frailty index and all-cause and cause-specific mortality in Chinese adults: a prospective cohort study. Lancet Public Health.

[27] Martín-Sánchez FJ, Fernández Alonso C, Perdigones J (2015). González del Castillo J: [Malnutrition: Another domain of frailty]. Med Clin (Barc).

[28] Rodríguez-Mañas L, Féart C, Mann G, Viña J, Chatterji S, Chodzko-Zajko W, Gonzalez-Colaço Harmand M, Bergman H, Carcaillon L, Nicholson C (2013). Searching for an operational definition of frailty: a Delphi method based consensus statement: the frailty operative definition-consensus conference project. J Gerontol A Biol Sci Med Sci.

[29] Sternberg SA, Wershof Schwartz A, Karunananthan S, Bergman H, Mark Clarfield A (2011). The identification of frailty: a systematic literature review. J Am Geriatr Soc.

[30] Morley JE, Vellas B, van Kan GA, Anker SD, Bauer JM, Bernabei R, Cesari M, Chumlea WC, Doehner W, Evans J (2013). Frailty consensus: a call to action. J Am Med Dir Assoc.

[31] Gobbens RJ, Luijkx KG, Wijnen-Sponselee MT, Schols JM (2010). Toward a conceptual definition of frail community dwelling older people. Nurs Outlook.

[32] Wei K, Nyunt MSZ, Gao Q, Wee SL, Ng TP (2017). Frailty and Malnutrition: Related and Distinct Syndrome Prevalence and Association among Community-Dwelling Older Adults: Singapore Longitudinal Ageing Studies. J Am Med Dir Assoc.

[33] Kizilarslanoglu MC, Sumer F, Kuyumcu ME (2016). Malnutrition increases frailty among older adults: How?. Clin Nutr.

[34] Artaza-Artabe I, Sáez-López P, Sánchez-Hernández N, Fernández-Gutierrez N, Malafarina V (2016). The relationship between nutrition and frailty: Effects of protein intake, nutritional supplementation, vitamin D and exercise on muscle metabolism in the elderly. A systematic review. Maturitas.

[35] Lilamand M, Kelaiditi E, Cesari M, Raynaud-Simon A, Ghisolfi A, Guyonnet S, Vellas B, van Kan GA (2015). Validation of the Mini Nutritional Assessment-Short Form in a Population of Frail Elders without Disability. Analysis of the Toulouse Frailty Platform Population in 2013. J Nutr Health Aging.

[36] Lopez D, Flicker L, Dobson A (2012). Validation of the frail scale in a cohort of older Australian women. J Am Geriatr Soc.

[37] de Vries NM, Staal JB, van Ravensberg CD, Hobbelen JS, Olde Rikkert MG (2011). Nijhuis-van der Sanden MW: Outcome instruments to measure frailty: a systematic review. Ageing Res Rev.

[38] Morley JE, Malmstrom TK, Miller DK (2012). A simple frailty questionnaire (FRAIL) predicts outcomes in middle aged African Americans. J Nutr Health Aging.

[39] Verlaan S, Aspray TJ, Bauer JM, Cederholm T, Hemsworth J, Hill TR, McPhee JS, Piasecki M, Seal C, Sieber CC (2017). Nutritional status, body composition, and quality of life in community-dwelling sarcopenic and non-sarcopenic older adults: A case-control study. Clin Nutr.

[40] OʼSúilleabháin PS, Gallagher S, Steptoe A (2019). Loneliness, Living Alone, and All-Cause Mortality: The Role of Emotional and Social Loneliness in the Elderly During 19 Years of Follow-Up. Psychosom Med.

[41] Liu H, Zhu D, Cao J, Jiao J, Song B, Jin J, Liu Y, Wen X, Cheng S, Nicholas S, Wu X (2019). The effects of a standardized nursing intervention model on immobile patients with stroke: a multicenter study in China. European journal of cardiovascular nursing: journal of the Working Group on Cardiovascular Nursing of the European Society of Cardiology.

[42] Liu H, Zhu D, Song B, Jin J, Liu Y, Wen X, Cheng S, Nicholas S, Wu X. Cost-effectiveness of an intervention to improve the quality of nursing care among immobile patients with stroke in China: a multicenter study. Int J Nurs Stud. 2020;110:103703. 10.1016/j.ijnurstu.2020.103703 32738722

[43] Chen CY, Wu SC, Chen LJ, Lue BH (2010). The prevalence of subjective frailty and factors associated with frailty in Taiwan. Arch Gerontol Geriatr.

[44] Si H, Jin Y, Qiao X, Tian X, Liu X, Wang C (2021). Predictive performance of 7 frailty instruments for short-term disability, falls and hospitalization among Chinese community-dwelling older adults: A prospective cohort study. Int J Nurs Stud.

[45] C JJYW. Z, F L, M Z, X W, J J, H W, D L, S Z, et al: Prevalence and associated factors for frailty among elder patients in China: a multicentre cross-sectional study. BMC Geriatr. 2020;20:100. 10.1186/s12877-020-1496-1 PMC7068995 32164595

[46] Lei Z, Qingyi D, Feng G, Chen W, Hock RS, Changli W (2009). Clinical study of mini-nutritional assessment for older Chinese inpatients. J Nutr Health Aging.

[47] Mahoney FI, Barthel DW (1965). Functional Evaluation: The Barthel Index. Md State Med J.

[48] Shiao CC, Hsu HC, Chen IL, Weng CY, Chuang JC, Lin SC, Tsai FF, Chen ZY (2015). Lower Barthel Index Is Associated with Higher Risk of Hospitalization-Requiring Pneumonia in Long-Term Care Facilities. Tohoku J Exp Med.

[49] Pascual JC, Belinchon I, Ramos JM (2015). Use of the Barthel index, activities of daily living, in dermatologic surgery in patients aged 80 years and older. Int J Dermatol.

[50] Lawton MP, Brody EM (1969). Assessment of older people: self-maintaining and instrumental activities of daily living. Gerontologist.

[51] Graf C (2008). The Lawton instrumental activities of daily living scale. Am J Nurs.

[52] Liang Y, Welmer AK, Moller J, Qiu C (2017). Trends in disability of instrumental activities of daily living among older Chinese adults, 1997–2006: population based study. BMJ Open.

[53] Ran L, Jiang X, Li B, Kong H, Du M, Wang X, Yu H, Liu Q (2017). Association among activities of daily living, instrumental activities of daily living and health-related quality of life in elderly Yi ethnic minority. BMC Geriatr.

[54] Arthur AJ, Jagger C, Lindesay J, Matthews RJ (2002). Evaluating a mental health assessment for older people with depressive symptoms in general practice: a randomised controlled trial. Br J Gen Pract.

[55] Chen D, Chen J, Yang H, Liang X, Xie Y, Li S, Ding L, Li Q (2019). Mini-Cog to predict postoperative mortality in geriatric elective surgical patients under general anesthesia: a prospective cohort study. Minerva Anestesiol.

[56] Chan CC, Fage BA, Burton JK, Smailagic N, Gill SS, Herrmann N, Nikolaou V, Quinn TJ, Noel-Storr AH, Seitz DP (2019). Mini-Cog for the diagnosis of Alzheimer’s disease dementia and other dementias within a secondary care setting. Cochrane Database Syst Rev.

[57] Chen LK, Woo J, Assantachai P, Auyeung TW, Chou MY, Iijima K, Jang HC, Kang L, Kim M, Kim S (2020). Asian Working Group for Sarcopenia: 2019 Consensus Update on Sarcopenia Diagnosis and Treatment. J Am Med Dir Assoc.

[58] Dent E, Lien C, Lim WS, Wong WC, Wong CH, Ng TP, Woo J, Dong B, de la Vega S, Hua Poi PJ (2017). The Asia-Pacific Clinical Practice Guidelines for the Management of Frailty. J Am Med Dir Assoc.

[59] Deschepper M, Eeckloo K, Vogelaers D, Waegeman W (2019). A hospital wide predictive model for unplanned readmission using hierarchical ICD data. Comput Methods Programs Biomed.

[60] Lim SL, Ong KCB, Chan YH, Loke WC, Ferguson M, Daniels L (2012). Malnutrition and its impact on cost of hospitalization, length of stay, readmission and 3-year mortality. Clin Nutr.

[61] Chumlea WC, Roche AF, Steinbaugh ML (1985). Estimating stature from knee height for persons 60 to 90 years of age. J Am Geriatr Soc.

[62] Latorre Román P, López DM, Aguayo BB, Fuentes AR, García-Pinillos F, Redondo MM (2017). Handgrip strength is associated with anthropometrics variables and sex in preschool children: A cross sectional study providing reference values. Phys Ther Sport.

[63] Beck AM, Kjær S, Hansen BS, Storm RL, Thal-Jantzen K (2011). Study protocol: follow-up home visits with nutrition: a randomised controlled trial. BMC Geriatr.

[64] Mitnitski AB, Mogilner AJ, Rockwood K (2001). Accumulation of deficits as a proxy measure of aging. ScientificWorldJournal.

[65] Rockwood K, Song X, MacKnight C, Bergman H, Hogan DB, McDowell I, Mitnitski A (2005). A global clinical measure of fitness and frailty in elderly people. CMAJ.

[66] Gobbens RJ, van Assen MA, Luijkx KG, Wijnen-Sponselee MT, Schols JM (2010). The Tilburg Frailty Indicator: psychometric properties. J Am Med Dir Assoc.

[67] Aygör HE, Fadıloğlu Ç, Şahin S, Aykar F, Akçiçek F (2018). Validation of Edmonton frail scale into elderly Turkish population. Arch Gerontol Geriatr.

