# Supplementary material for: An early predictive model of frailty for older inpatients according to nutritional risk: protocol for a cohort study in China
Source: BMC Geriatr. 2021 Aug 18;21:465. doi: 10.1186/s12877-021-02396-3 (PMC8371757; doi:10.1186/s12877-021-02396-3)
Supplement: Supplementary file 1 — Additional file 1. [file 12877_2021_2396_MOESM1_ESM.pdf]

B2019001051

Case Report Form

Develop and application an early predictive model of frailty for older inpatients according to nutritional risk: a cohort study in China

Name：

Medical record number：

Department：

Chinese Academy of Medical Sciences - Peking Union Medical College, Peking Union Medical College Hospital

B2019001051

Content

Inclusion and exclusion criteria 1

Form 1. Demographic characterics（Complete assessment within 48 hours of admission） 3

Form 2. Scale assesssment（Complete assessment within 48 hours of admission） 5

Form 3.Outcomes 7

Form 4.Follow-up（at 30-day follow-up） 7

Inclusion and exclusion criteria

**Medical record number**: ______ **Bed number**: _________<br>**Name**______		
**Date of admission**: ____Day ____Month ____Year<br>**Date of birth**: ____Day ____Month ____Year		
**inclusion and exclusion criteria**		
**inclusion**: age 65 years or more	□Yes	□No
**inclusion**: estimated survival time > 3 months	□Yes	□No
**inclusion**: hospitalized for minimum of 4 days in the wards of Peking Union Medical College Hospital	□Yes	□No
**inclusion**: BI （Barthel Index） scores ≥ 75 points (BI use 1.a to assess, see 1.a below please)	□Yes	□No
**inclusion**: malnourished or at risk of malnutrition according to the Mini-Nutritional Assessment-Short Form (MNA-SF; scores range from 0 to 11) (MNA-SF use 1.b to assess, see 1.b below please)	□Yes	□No
**inclusion**: provid written informed consent	□Yes	□No
**exclusion**: patients who are persistently unconsciousness or unable to provide informed consent for participation, or their caregivers were unable to provide effective information	□Yes	□No
**exclusion**: patients with anorexia nervosa, acute pancreatitis, acute liver failure, cystic fibrosis, stem cell transplantation, severe chronic gastrointestinal diseases, acute infectious diseases or chronic wasting diseases at enrollment	□Yes	□No
**exclusion**: patients who were initially admitted to the intensive care unit (ICU)	□Yes	□No
**exclusion**: patients who were included in model development	□Yes	□No

B2019001051

during the first stage		

1.a Barthel Index

number	items	Independent (points)	Minor help (points)	Major help (points)	Dependent (points)
1	feeding	10	5	0	-
2	Bathing	5	0	-	-
3	Grooming	5	0	-	-
4	Dressing	10	5	0	-
5	Bowels	10	5	0	-
6	Bladder	10	5	0	-
7	Toilet use	10	5	0	-
8	Transfers (bed to chair and back)	15	10	5	0
9	Mobility (on level surfaces)	15	10	5	0
10	Stairs	10	5	0	-

Total score： ____

1.b Nutritional status

A. Has food intake declined over the past 3 months due to loss of appetite, digestive problems, chewing or swallowing difficulties?	□ severe decrease in food intake (＞75%)（0 points）<br>□ moderate decrease in food intake（1 points）<br>□ no decrease in food intake（≤10%）（2 points）
B. Weight loss during the last 3 months.	□ weight loss greater than 3 kg（0 points）

B2019001051

B2019001051

	□ does not know （1 points）<br>□ weight loss between 1 and 3 kg （2 points）<br>□ no weight loss （3 points）
C. Mobility	□ bed or chairbound （0 points）<br>□ able to get out of bed/chair but does not go out（1 points）<br>□ goes out（2 points）
D. Has suffered psychological stress or acute disease in the past 3 months?	□ yes （0 points）<br>□ no（2 points）
E. Neuropsychological problems	□ severe dementia or depression（0 points）<br>□ mild dementia （1 points）<br>□ no psychological problems（2 points）
F1. Body mass index (BMI) (weight in kg)/(height in m^2)	□BMI <19 （0 points）<br>□19≤BMI＜21（1 points）<br>□21≤BMI＜23（2 points）<br>□BMI≥23 （3 points）
F2 Calf circumference（cc）in cm (IF BMI IS NOT AVAILABLE, REPLACE QUESTION F1 WITH QUESTION F2)	□cc <31（0 points）<br>□cc≥31（3 points）

Total score _______

Form 1. Demographic characterics（Complete assessment within 48 hours of admission）

A1 **Main diagnosis**：1.________ 2.________ 3._________4.________ 5.______

A2 **Sex**： □Male □Female

A3 **ethnicity**：□Han □ethnic minorities:________

A4 **Education level**：□illiterate □primary school □junior high school □high school and above □others：_______

A5 **Marital status**：□married □divorced or widowed □others：_______

A6 **Home address**：____（Province）____（City）____(District)_________

B2019001051

A7 **Patient phone number：** __________ **patients' family members phone number：** __________

A8 **Height：** ___cm **Weight：** ____kg ，□ Can not be measured：**Calf circumference (cc)**________ cm

A9 **Admission to the hospital:** □ outpatient department □ emergency department □ transit from other hospitals □ others______

A10 **Type of insurance：**

□ New Cooperative Medical System □ Urban Resident Basic Medical Insurance

□ Urban Employee Basic Medical Insurance

□ Others_______

A11 **Living alone：** □Yes □No（for example：living with children, living with a spous, living with a nanny, living in a nursing home, others_______

A12 **Living conditions：** □ living in a building with an elevator □ living in a building without an elevator □ bungalow

A13 **Smoking：**

□ non-smoker □ current smoker □ former smoker

A14 **Alcohol consumption：**

□ non-drinker □ current drinker □ former drinker

A15 **Falling accidents in the past 12 months:** □No □Yes，___times

A16 **Immobilization for more than 4 weeks** □No □Yes

A17 **Polypharmacy：** □No □Yes：（Number of medications：___）

A18 **Whether a blood sample was taken：** □Yes：Inspection nmber______

□No：（reason：______）

A19 **handgrip strength：** ________kg □ Can not be measured，reason：_______

B2019001051

A20 **Vision：**□ Normal □Dysfunction

A21 **Hearing** □ Normal □ Dysfunction

A22 **Sleeping：**□ Normal □ Dysfunction（for example：Difficulty falling asleep, frequent awakening, early awakening, excessive daytime sleepiness, taking sleeping pills, others____________）

A23 **Urinary function** □Normal □ Dysfunction（Such as: frequent urination, urinary incontinence, indwelling catheter, dysuria, urinary retention, others________）

A24 **Defecation function** □Normal □ Dysfunction（Such as: constipation, diarrhea, fecal incontinence, fistula, others_______）

A25 **Pain：**□No □Yes：（NRS score： ______scores）

Form 2. Scale assesssment（Complete assessment within 48 hours of admission）

2.1 New early predictive model of frailty for older inpatients according to nutritional risk

2.2 Fried Phenotype

Number	Items	Male	Female
1	Weight loss	weight loss, unintentional, of > 3kg in prior year or, at follow-up, of >5% of body weight in prior year (by direct measurement of weight).	
2	Exhaustion	Answer "Yes" to one of the following 2 questions (more than three days in the last week):<br>(a) I felt that everything I did was an effort<br>(b) I could not get going	
3	Grip Strength	BMI≤24.0 kg/m^2： ≤29<br>BMI 24.1~26.0 kg/m^2: ≤30<br>BMI 26.1~28.0 kg/m^2:≤30<br>BMI＞28 kg/m$^{2/:}$ ≤32	BMI≤23.0 kg/m^2： ≤17<br>BMI 23.1~26.0 kg/m^2:≤17. 3<br>BMI 26.1~29.0 kg/m2:≤18<br>BMI＞29.0 kg/m^2 :≤21

4	Walk Time（4.5m)	Height≤173 cm:≥7 s;<br>Height＞173 cm:≥6 s	Height≤159 cm:≥7 s;<br>Height＞159 cm:≥6 s
5	Physical activity	＜383 kcal/week (Walking about 2.5 h)	＜270 Kcal/week (Walking about 2.0 h)

2.3 FRAIL Scale

Assessment	Items	Content
□Yes □No	Fatigue	During the past 4 weeks they felt tired with responses of “all of the time“ or “most of the time”
□Yes □No	Resistance	Had any difficulty walking up 10 steps alone without resting and without aids
□Yes □No	Ambulation	Had any difficulty walking several hundred yards alone and without aids
□Yes □No	Illnesses (>5)	Respondents who reported 5 or more illnesses out of 11 total illnesses (hypertension, diabetes, acute heart disease, stroke, malignant tumors (except small skin cancer), congestive heart failure, asthma, arthritis, chronic lung disease, kidney disease, angina pectoris)
□Yes □No	Loss of weight >5%	Respondents with a weight decline of 5% or greater within the past 12 months based on self-report

Total score： ___scores （Choose "Yes" to get 1 point, 5 points in total.）

B2019001051

B2019001051

Form 3. Outcomes

3.1 Outcomes：

□ **Discharged：** ______; date of discharged： ____Day ____Month ____Year

□ **Transferred to other departments：** department： ______; main diagnosis：

1.________2. ________ 3. ________4.________5. ________； date of transferred：

____Day ____Month ____Year

□ **Death** date of death： ____Day ____Month ____Year

□ **Others：** □ Abandon treatment □ Medical disputes □ Others： ___________

Date: ____Day ____Month ____Year

3.2 Does the patient fall or fall from bed during hospitalization?

□ No □ falls，date: ____Day ____Month ____Year

□ fall from bed，date: ____Day ____Month ____Year

3.3 Was the patient transferred to the intensive care unit during the hospital stay?

□No □Yes： _______days

date of transferred in： ____Day ____Month ____Year;

date of transferred out： ____Day ____Month ____Year

Form 4. Follow-up（at 30-day follow-up）

Name： ____ **; date of enrollment：** ____Day ____Month ____Year

4.1 Follow-up results: □ Complete follow-up

□ lost to follow-up

□ decline to follow-up

□ Death： ____Day ____Month ____Year reason:_________

□ Others： _________

The person being followed-up： □ patient □ family members □ Nanny and other non-relative care workers

4.2 Patient's current residence： □ home □ nursing homes or welfare institutions □

B2019001051

hospitals

4.3 Patient's medical visits during follow-up： □ No □Yes

□ outpatient department, number of visits： ____times

□ emergency department, number of visits： ____times

□ hospital admission： length of hospital stay____days

Reason for readmission □ check the effect of treatment □ prescribe medicine

□ chemotherapy □ others ______

4.4 Have you ever fallen or fallen from bed since you were discharged from the hospital： □No □ Falls： ____times：（Date： ____Day ____Month ____Year）;

□ fall from bed，: ____times：（Date：____Day ____Month ____Year）

4.5 Did you have any experience in intensive care unit treatment after discharge： □No □Yes：（reason： ______）

4.6 Whether the following complications occured：

1. adjudicated diagnosis of nosocomial infection：□No □Yes：（Date： ____Day ____Month ____Year）;

2.respiratory failure： □No □Yes：（Date： ____Day ____Month ____Year）;

3.cardiovascular event [for instance： stroke： □No □Yes：（Date： ____Day ____Month ____Year）; intracranial bleeding： □No □Yes：（Date： ____Day ____Month ____Year）; cardiac arrest： □No □Yes：（Date： ____Day ____Month ____Year）; myocardial infarction： □No □Yes：（Date： ____Day ____Month ____Year）; pulmonary embolism： □No □Yes：（Date： ____Day ____Month ____Year）];

4. acute renal failure： □No □Yes：（Date： ____Day ____Month ____Year）;

5. gastrointestinal failure， i.e., hemorrhage, intestinal perforation, acute pancreatitis： □No □Yes：（Date： ____Day ____Month ____Year）

4.7 Frailty assessment

If applicable： □Yes（fill in the form below） □ not applicable（reason： □unconscious □others： _____)

4.7.1 New early predictive model of frailty for older inpatients according to nutritional risk

4.7.2 Fried Phenotype

Number	Items	Male	Female
1	Weight loss	weight loss, unintentional, of > 3kg in prior year or, at follow-up, of >5% of body weight in prior year (by direct measurement of weight).	
2	Exhaustion	Answer "Yes" to one of the following 2 questions (more than three days in the last week):<br>(a) I felt that everything I did was an effort<br>(b) I could not get going	
3	Grip Strength	BMI≤24.0 kg/m^2： ≤29<br>BMI 24.1~26.0 kg/m^2: ≤30<br>BMI 26.1~28.0 kg/m^2:≤30<br>BMI＞28 kg/m$^{2/:}$ ≤32	BMI≤23.0 kg/m^2： ≤17<br>BMI 23.1~26.0 kg/m^2:≤17. 3<br>BMI 26.1~29.0 kg/m2:≤18<br>BMI＞29.0 kg/m^2 :≤21
4	Walk Time（4.5m)	Height≤173 cm:≥7 s;<br>Height＞173 cm:≥6 s	Height≤159 cm:≥7 s;<br>Height＞159 cm:≥6 s
5	Physical activity	＜383 kcal/week (Walking about 2.5 h)	＜271 Kcal/week (Walking about 2.0 h)

4.7.3 Frail Scale

Assessment	Items	Content
□Yes □No	Fatigue	During the past 4 weeks they felt tired with responses of “all of the time“ or “most of the time”
□Yes □No	Resistance	Had any difficulty walking up 10 steps alone without resting and without aids
□Yes □No	Ambulation	Had any difficulty walking several hundred yards alone and without aids
□Yes □No	Illnesses (>5)	Respondents who reported 5 or more illnesses out of 11 total illnesses (hypertension, diabetes, acute heart disease, stroke,

B2019001051

		malignant tumors (except small skin cancer), congestive heart failure, asthma, arthritis, chronic lung disease, kidney disease, angina pectoris)
□Yes □No	Loss of weight >5%	Respondents with a weight decline of 5% or greater within the past 12 months based on self-report

Total score： ___scores （Choose "Yes" to get 1 point, 5 points in total.）

4.8 Barthel index

number	items	Independent (points)	Minor help (points)	Major help (points)	Dependent (points)
1	feeding	10	5	0	-
2	Bathing	5	0	-	-
3	Grooming	5	0	-	-
4	Dressing	10	5	0	-
5	Bowels	10	5	0	-
6	Bladder	10	5	0	-
7	Toilet use	10	5	0	-
8	Transfers (bed to chair and back)	15	10	5	0
9	Mobility (on level surfaces)	15	10	5	0
10	Stairs	10	5	0	-

B2019001051

B2019001051

Total score：_____

4.9 Quality of life

If applicable：□Yes（） □ not applicable（reason：□unconscious □others：_____)

number	items	options
1	Mobility	□ have no problems/be not<br>□ have some/moderate problems<br>□have extremely problems/unable to
2	Self-care	□ have no problems/be not<br>□ have some/moderate problems<br>□have extremely problems/unable to
3	Usual activities	□ have no problems/be not<br>□ have some/moderate problems<br>□have extremely problems/unable to
4	Pain/discomfort	□ have no problems/be not<br>□ have some/moderate problems<br>□have extremely problems/unable to
5	Anxiety/depression	□ have no problems/be not<br>□ have some/moderate problems<br>□have extremely problems/unable to
6	Self-reported health states (the best state you can imagine is marked 100 and the worst state you can imagine is marked 0)	____ scores
